# Physical activity in relation to irritable bowel syndrome among Iranian adults

**DOI:** 10.1371/journal.pone.0205806

**Published:** 2018-10-18

**Authors:** Mehdi Sadeghian, Omid Sadeghi, Ammar Hassanzadeh Keshteli, Hamed Daghaghzadeh, Ahmad Esmaillzadeh, Peyman Adibi

**Affiliations:** 1 Food Security Research Center, Isfahan University of Medical Sciences, Isfahan, Iran; 2 Department of Community Nutrition, School of Nutrition and Food Science, Isfahan University of Medical Sciences, Isfahan, Iran; 3 Students' Scientific Research Center, Tehran University of Medical Sciences, Tehran, Iran; 4 Department of Community Nutrition, School of Nutritional Sciences and Dietetics, Tehran University of Medical Sciences, Tehran, Iran; 5 Department of Medicine, University of Alberta, Edmonton, AB, Canada; 6 Integrative Functional Gastroenterology Research Center, Isfahan University of Medical Sciences, Isfahan, Iran; 7 Obesity and Eating Habits Research Center, Endocrinology and Metabolism Molecular -Cellular Sciences Institute, Tehran University of Medical Sciences, Tehran, Iran; Howard University, UNITED STATES

## Abstract

**Background:**

Irritable bowel syndrome (IBS) is the most prevalent functional gastrointestinal disorder worldwide. Physical activity in relation to IBS has been investigated in few studies and data in this regard are conflicting.

**Aim:**

To investigate the association between physical activity and IBS in a large sample of Iranian adults.

**Methods:**

This cross-sectional study was done on 4763 Iranian adults in the framework of SEPAHAN (The Study on the Epidemiology of Psycho-Alimentary Health and Nutrition) project. The physical activity of study participants was assessed using the General Practice Physical Activity Questionnaire (GPPAQ). Using a validated self-administered modified Rome III questionnaire, functional gastrointestinal disorders including irritable bowel syndrome was assessed.

**Results:**

The mean age of study participants was 36.5 years. Irritable bowel syndrome was prevalent among 21.5% of participants. Compared with physically active individuals (≥ 1 hour/wk), those with sedentary physical activity (<1 hour/wk) had 1.27 times greater probability of suffering from IBS (OR: 1.27, 95% CI: 1.08–1.49). However, this association was attenuated after adjusting for age, sex, cigarette smoking and medical history of colitis and diabetes. When the analysis was additionally adjusted for diet-related practices and body mass index (BMI), a non-significant association was found between sedentary physical activity and IBS (OR: 1.18, 95% CI: 0.98–1.41). Gender-stratified analysis revealed similar findings in women either before (OR: 1.29, 95% CI: 1.04–1.61) or after controlling for covariates (OR: 1.27, 95% CI: 0.99–1.62). In BMI-stratified analysis, a significant positive association was seen between sedentary physical activity and IBS among individuals with normal BMI (OR: 1.38, 95% CI: 1.07–1.79).

**Conclusion:**

We found a significant positive association between sedentary physical activity and IBS, particularly among women and individuals of normal weight.

## Introduction

Irritable bowel syndrome (IBS) is a chronic condition characterized by recurrent abdominal pain or discomfort associated with disturbances in defecation, stool frequency or stool form [1]. Different diagnostic criteria used in earlier studies have resulted in various ranges of prevalence of this syndrome [2]. It is highly prevalent globally and varies from 5–10% in Asia to 9–22% in the European countries [3–5]. In Iran, its prevalence has been estimated to vary from 1.1% to 25% in various studies [6]. Patients suffering from IBS have unfavorable quality of life and considerable costs induced due to subsequent medical care [7].

Several factors, including environmental factors, contribute to IBS etiology [8]. Physical activity is of benefit for health; however, limited data with conflicting results are available linking physical activity to IBS. Previous studies have reported that spending more time doing moderate physical activity is associated with improved symptoms of IBS [9]. In healthy individuals, physical activity has been associated with improvement in gas transit and abdominal distension, symptoms that are frequently observed in IBS patients [10]. Other medical conditions including depression and fibromyalgia, which accompany with IBS, were suggested to undergo reduction with regular mild exercise [11]. Other reports have also documented the beneficial role of physical activity in IBS management [12–13]. Findings from case-control studies have revealed lower physical activity levels in patients with IBS [14, 15]. However, some investigators have failed to find a significant association between physical activity and IBS. Omagari et al [8] reported a high level of physical activity among patients with IBS than those without IBS. Overall, it seems that further studies are required to shed light on this association. It must be kept in mind that almost all earlier studies have been confined to IBS symptoms rather than its prevalence as a functional gastrointestinal disorder and we are aware of no epidemiologic study investigating the association between physical activity and IBS [11–13, 16, 17]. In addition, previous observational studies were conducted among a group of IBS patients and no population-based study is available in this regard. Furthermore, almost all prior investigations were performed on a small sample size and those with relatively large samples have been confined to children. Moreover, all earlier studies were conducted in Western countries and, to the best of our knowledge, no such report is available from Middle-Eastern countries. Considering the cultural differences between the Middle-Eastern and Western populations, the association between physical activity and IBS might be different in this part of the world. In the current study, we aimed to investigate the association of physical activity with IBS in the framework of a large population-based study in Iran.

## Subjects and methods

### Participants

We used data from SEPAHAN (The Study on the Epidemiology of Psycho-Alimentary Health and Nutrition) project to conduct this cross-sectional study. This project, which was conducted in 2010, investigated the epidemiological aspects of functional gastrointestinal disorders and their relationships with different lifestyle factors. Study design, sampling methods, participant characteristics and data collection procedures have been previously published in detail [18]. Staff from Isfahan University of Medical Sciences (IUMS) working in 50 different centers (health centers, hospitals, administrative sections) were included in the current study. Data was collected in two separate phases. In the first phase, 10087 adults were asked to complete self-administered questionnaires on lifestyle factors, including physical activity with 8691 subjects returning the completed questionnaire (response rate: 86.2%). In the second phase, 9652 questionnaires containing information on gastrointestinal symptoms were sent to the participants and 6239 completed questionnaires were returned (response rate: 64.6%). When we combined data from both phases, information was available for 4763 subjects who had completed both questionnaires. All data was fully anonymized before the researcher had access. Signed written consent forms were provided by all participants. The study was approved by Isfahan University of Medical Sciences, Isfahan, Iran (www.mui.ac.ir) (projects numbers #189069, #189082, and #189086).

### Assessment of physical activity

The physical activity levels of study participants were assessed using the General Practice Physical Activity Questionnaire (GPPAQ). This questionnaire is a simple tool ranking participants’ physical activity with focus on their current general activities. GPPAQ is a validated short measure of physical activity that was accredited by the Department of Health and developed by the London School of Hygiene and Tropical Medicine [19]. Patients were requested to report their activities based on GPPAQ, which took approximately 30 seconds to fill. Current physical activity has been used as a reliable contributor for objective assessment of overall physical activity levels. Based on their responses, participants were classified into 4 categories; 1) Inactive (sedentary job and no physical exercise or cycling), 2) Moderately inactive (sedentary job and some but <1 hour physical exercise and/or cycling per week OR standing job and no physical exercise or cycling), 3) Moderately active (sedentary job and 1–2.9 hours physical exercise and/or cycling per week OR standing job and some but <1 hour physical exercise and/or cycling per week OR physical job and no physical exercise or cycling), 4) Active (sedentary job and > 3 hours physical exercise and/or cycling per week OR standing job and 1–2.9 hours physical exercise and/or cycling per week OR physical job and some but < 1 hour physical exercise and/or cycling per week OR heavy manual job). However, in the current study due to low number of subjects in some of the above-mentioned categories, individuals in the “inactive” and “moderately inactive” groups were combined and were defined as those with “sedentary physical activity”. Similarly, individuals in the “moderately active” and “active” categories were combined and then defined as “physically active”.

### Assessment of irritable bowel syndrome

Using a validated self-administered modified Rome III questionnaire, different gastrointestinal disorders including symptoms related to IBS were assessed [1]. Since during the face validity evaluation of our study, we found that most participants found it difficult to discriminate the descriptors used in the original Rome III (never, less than one day a month, one day a month, two to three days a month, one day a week, more than one day a week, every day), we modified the rating scale into 4 descriptors (i.e. never or rarely, sometimes, often, always). We also asked whether each symptom was present in the past three months. In the current study, on the basis of the Rome III diagnostic criteria for functional gastrointestinal disorders, we defined IBS as having recurrent abdominal pain or discomfort at least “sometime” in the last 3 months associated with two or more of these criteria: improvement with defecation at least “sometimes” and onset associated with change in frequency or form (appearance) of stool at least “sometimes”. The Persian version of the Rome III questionnaire for Iranian population was validated previously [20].

### Assessment of other variables

Information on age, gender, weight, height, smoking status (non-smokers, ex-smokers and current smokers) and educational status were collected using a self-reported questionnaire. Furthermore, a questionnaire was used to gather data on medical history (diabetes and colitis) and medications used for IBS treatment. Participants were questioned regarding meal regularity (never/sometimes/often/always), chewing sufficiency (a lot/moderately/little), eating rate (<10 min/≥10 min), intra-meal fluid consumption (never/sometimes/often/always) as well as the frequency of breakfast consumption (never or 1 day per week/2–4 days per week/5–6 days per week and every day), spicy food intake (never, 1–3 times/wk, 4–6 times/wk or more than 10 times), quantity of consumed spices (low, moderate, high), and fried food consumption (never, 1–3 times/wk, 4–6 times/wk or every day). In terms of dental status, participants were classified into 3 categories; “fully dentate”, “loss of 1–5 teeth” and “loss of more than 5 teeth”.

### Statistical analysis

All statistical analyses were done in SPSS (version 18). We applied independent samples t-test to examine significant differences between participants with sedentary physical activity and physically active in terms of continuous variables. Distribution of participants with regards to categorical variables was evaluated using Chi-square test. To examine the odds of IBS among categories of physical activity, we used binary logistic regression in crude and adjusted models. In the first model, age (continuous) and gender (categorical) were adjusted for. Further controlling was done for cigarette smoking and medical history (colitis, diabetes) in the second model. Additional adjustment was done for regular meal pattern (never/sometimes/often/always), eating rate (<10 min/≥10 min), chewing sufficiency (a lot/moderately/little), breakfast consumption (never or 1 day per week/2–4 days per week/5–6 days per week and every day), intra-meal fluid consumption (never/sometimes/often/always), fried food intake (never/1–3 times per week/4–6 times per week or every day), tooth status (have all teeth/lost 1–5 teeth/lost >5 teeth) and spicy food intake (never, 1–3 times/wk, 4–6 times/wk or more than 10 times) in the third model. In the final model, body mass index (BMI) (continuous) was additionally adjusted for to see if the association between physical activity and IBS was independent of obesity. In all mentioned analyses, physically active participants were considered as reference. The analysis, with the same covariates as mentioned above, was also done separately by gender and categories of BMI (<25/≥25 kg/m^2^). In all statistical analyses, P values of less than 0.05 were considered as significant.

## Results

The mean age of study participants was 36.5±8.0 years. IBS was prevalent among 21.5% (n = 1024) of participants. General characteristics of study participants by categories of physical activity are described in **Table 1**. Compared with physically active individuals, those with sedentary physical activity were younger, weighed less and were less likely to be male, have regular meal pattern, chew their foods sufficiently and were more likely to consume fluids during a meal.

**Table 1:** General characteristics of study participants by categories of physical activity.

	physically active^1^	sedentary physical activity^2^	P-value
Age (y)	36.8 ± 8.32	36.0 ± 7.78	0.01
Weight (kg)	70.6 ± 12.71	67.8 ± 12.58	<0.001
BMI (kg/m^2^)	24.8 ± 3.66	24.9 ± 3.95	0.50
Male (%)	56.5	36.5	<0.001
Current smokers (%)	13.2	13.5	0.09
Diabetes (%)	1.6	1.8	
Colitis (%)	1.0	1.2	
Regular meal pattern^3^ (%)	62.9	58.1	0.01
Chewing sufficiency (a lot) (%)	16.8	12	<0.001
Fluid consumption (always) (%)	22.2	25.6	0.001
Breakfast skipping^4^ (%)	20.1	23.7	0.06
Frequent fried food intake^5^ (%)	16.2	16.9	0.08
Tooth loss (Have all) (%)	29.6	34.4	0.07

^1^ defined as individuals with ≥1 hour/week physical activity

^2^defined as individuals with <1 hour/week physical activity

^3^ defined as individuals who have reported having regular meals often or always

^4^ defined as individuals who were eating breakfast < 5 times/week

^5^ defined as individuals who consumed fried foods ≥ 4 times/week

Multivariable-adjusted odds ratios (95% CIs) for IBS across categories of physical activity levels are provided in **Table 2**. Those with sedentary physical activity had 1.27 times greater probability of suffering from IBS compared with physically active individuals (OR: 1.27, 95% CI: 1.08–1.49). However, this association was attenuated after adjusting for age, sex, cigarette smoking and medical history of colitis and diabetes. When the analysis was additionally adjusted for diet-related practices and BMI, this association became non-significant (OR: 1.18, 95% CI: 0.98–1.41). We failed to find any significant association between physical activity and IBS in men. However, among women, we found a positive significant association between sedentary physical activity and odds of IBS; such that after controlling for age, sex, cigarette smoking and medical history of colitis and diabetes, women with sedentary physical activity were 29% more likely to have IBS than physically active individuals (OR: 1.29, 95% CI: 1.02–1.63). However, this relationship became non-significant after additional adjustment for diet-related practices and BMI (OR: 1.27, 95% CI: 0.99–1.62). When we did the analyses stratified by different diet-related practices, again we failed to find any significant association.

**Table 2:** Multivariable-adjusted odds ratios (95% CIs) for irritable bowel syndrome across categories of physical activity levels^1^.

		Categories of physical activity	
		physically active^2^	sedentary physical activity^3^
Whole population			
	Crude	1.00	1.27 (1.08–1.49)
	Model 1	1.00	1.20 (1.01–1.43)
	Model 2	1.00	1.19 (1.01–1.42)
	Model 3	1.00	1.18 (0.98–1.41)
	Model 4	1.00	1.18 (0.98–1.41)
Men			
	Crude	1.00	1.10 (0.87–1.40)
	Model 1	1.00	1.09 (0.84–1.42)
	Model 2	1.00	1.08 (0.84–1.41)
	Model 3	1.00	1.11 (0.84–1.47)
	Model 4	1.00	1.11 (0.84–1.47)
Women			
	Crude	1.00	1.29 (1.04–1.61)
	Model 1	1.00	1.31 (1.04–1.65)
	Model 2	1.00	1.29 (1.02–1.63)
	Model 3	1.00	1.27 (0.99–1.62)
	Model 4	1.00	1.27 (0.99–1.62)

^1^ Values are ORs (95% CIs).

IBS was defined as having recurrent abdominal pain or discomfort at least sometime in the last 3 months associated with two or more of these criteria: improvement with defecation at least sometimes and onset associated with change in frequency or form (appearance) of stool at least sometimes

^2^ defined as individuals with ≥1 hour/week physical activity

^3^ defined as individuals with <1 hour/week physical activity

Model 1: Adjusted for age, sex

Model 2: Additionally adjusted for cigarette smoking, medical history (colitis, diabetes)

Model 3: Further adjusted for regular meal pattern, eating rate, chewing sufficiency, breakfast consumption, fluid consumption during a meal, fried food intake, tooth status and spicy food intake

Model 4: Additionally adjusted for BMI

BMI-stratified analysis on the association between physical activity and IBS is shown in **Table 3**. Among individuals with normal weight, those with sedentary physical activity had 35% greater odds of IBS than physically active participants (OR: 1.35, 95% CI: 1.08–1.68). This association remained significant even after taking potential confounders into account (OR: 1.38, 95% CI: 1.07–1.79). Among overweight or obese individuals, no significant association was seen between physical activity and odds of IBS.

**Table 3:** Multivariable-adjusted odds ratios (95% CIs) for irritable bowel syndrome across categories of physical activity levels stratified by BMI status^1^.

		Categories of physical activity	
		physically active^2^	sedentary physical activity^3^
BMI<25			
	Crude	1.00	1.35 (1.08–1.68)
	Age-adjusted	1.00	1.34 (1.06–1.69)
	Multivariable-adjusted^4^	1.00	1.38 (1.07–1.79)
BMI≥25			
	Crude	1.00	1.16 (0.92–1.47)
	Age-adjusted	1.00	1.17 (0.91–1.49)
	Multivariable-adjusted^4^	1.00	1.00 (0.76–1.32)

^1^ Values are ORs (95% CI).

IBS was defined as having recurrent abdominal pain or discomfort at least sometime in the last 3 months associated with two or more of these criteria: improvement with defecation at least sometimes and onset associated with change in frequency or form (appearance) of stool at least sometimes

^2^ defined as individuals with ≥1 hour/week physical activity

^3^ defined as individuals with <1 hour/week physical activity

^4^ Adjusted for age, gender, cigarette smoking, medical history (colitis, diabetes), regular meal pattern, eating rate, chewing sufficiency, breakfast consumption, fluid consumption during a meal, fried food intake, tooth status and spicy food intake

## Discussion

In the current study, we found a significant inverse association between physical activity and risk of IBS; individuals with sedentary physical activity had 27% greater odds of having IBS compared with physically active individuals. However, this was not significant after controlling for diet related practices and BMI. In addition, sex-stratified analysis revealed no significant association in men and women in fully adjusted model. We also found a significant positive association between sedentary activity and IBS among individuals of normal weight when potential confounders were taken into account. Although the sample size of the study was high, the obtained effect sizes for the association between physical activity and IBS were small. To the best of our knowledge, the current study was the first to examine the association between physical activity and IBS in the Middle East.

The prevalence of IBS in the current study is in line with earlier reports from Asian countries [21, 22]; however, it is much higher than global prevalence due to the use of a modified Rome III criteria rather than a clinical assessment for identification of IBS [23]. IBS is associated with several disabilities and significant incurred costs. Among environmental factors contributing to IBS, less attention has been paid on physical activity. Based on our findings, individuals with sedentary activity have greater risk of IBS. In line with our findings, in a cross-sectional study by “Kim et. al”, using a self-reported questionnaire applied with a large population of university students, physical activity was inversely associated with IBS [16].” Lustyk et. al” reported that patients with IBS had spent less time on exercise compared with healthy individuals [12]. Findings from a case-control study revealed that physically active subjects were 3.6 times less likely to suffer from IBS than those with a physically inactive lifestyle [24]. Moreover, a weak trend was observed toward higher prevalence of IBS with fewer hours spent for physical activity (odds ratio: 0.99) [16]. In a clinical trial on patients with IBS, a 12-week regiment of moderate activity in the intervention group improved gastrointestinal symptoms and quality of life [13]. Altogether, it seems that promoting physical activity in the general population may help prevention of developing IBS. When we controlled for the effect of dietary habits, the association between sedentary activity and IBS became non-significant. It seems that positive association between sedentary physical activity and IBS are mediated by dietary habits or diet-related practices. For example, it has been shown that sedentary individuals consume more fluid during a meal than active ones [25]. As seen in our study, breakfast skipping, having an irregular meal pattern and chewing insufficiency were common in sedentary individuals compared with physically active ones. All mentioned diet-related practices are positively associated with IBS [25]. Therefore, this could be an explanation for the non-significant association after controlling dietary practices.

In the current study, we observed a significant positive association between sedentary physical activity and IBS among normal-weight individuals, but not among those who were overweight or obese. This difference might be explained by the effects of obesity on the regulation of gastrointestinal hormones in obese individuals compared with normal weight subjects [26]. In addition, a recent meta-analysis revealed the different effects of physical activity on secretion of Glucagon-like peptide 1 (GLP-1) in normal-weight individuals compared with those with overweight or obesity. Since GLP-1 is implicated in the pathogenesis of IBS [27], different effects of physical activity on GLP-1 may be another reason for positive association between sedentary physical activity and IBS among normal-weight individuals, but not in those with overweight or obesity. Furthermore, a clinical trial showed that psychological stress affect less individuals with overweight or obesity than those with normal weight [28]. As reported previously, psychological stress is a risk factor for IBS.

The mechanisms behind the association between physical activity and IBS are unknown. It may be explained by the change of gas transit and colonic transit due to increased physical activity [10]. Moreover, physical activity can favorably influence brain plasticity by facilitating neurogenerative, neuroadaptive, and neuroprotective processes [29], thus have a positive effect on the brain-gut axes which is involved in IBS.

This study has several strengths. This is the first study investigating the association between IBS prevalence and physical activity in a large sample of adults. In the current study we controlled the analyses for a wide range of confounders including dietary habits which were not taken into account in earlier studies. In addition, a large sample size enabled us to conduct stratified analysis by gender and BMI categories, which was not done previously. A major weakness of this study is the cross-sectional design that limits causal inferences. In addition, the observed associations might be attributed to other factors including high intake of spicy or fatty foods [30]. Moreover, the results might not be extrapolated to the general population of Iran living in other provinces as the sampled population was a group of adults affiliated to a medical University. Although several confounders were adjusted to assess the association between physical activity and IBS, some residual confounders including psychological disorders and drug or supplements use might affect the obtained risk estimates. Furthermore, both physical activity and IBS prevalence (21.5%) were examined through the use of questionnaires in this study. However, it should be noted that the Rome III questionnaire for assessment of gastrointestinal health has been validated in the Iranian population previously and the diagnosis of IBS is criteria-based and does not require clinical assessment by a gastroenterologist in large-scale epidemiological studies. Although, the validity of GPPAQ has earlier been examined in several populations, its validity and reliability has not been tested in Iran. Therefore, misclassification of participants may be another problem in the current study. Moreover, inability to separately analyze for activity at work or leisure time is another limitation of our study. While physical activity at work appears to exacerbate gastrointestinal symptoms, leisure time activity may be a protective factor [31].

In conclusion, we found a marginally significant positive association between sedentary physical activity and IBS, particularly among women and individuals of normal weight. Further studies, particularly of prospective design, are needed to confirm our findings.
